# Supplementary material for: Sporadic ALS Astrocytes Induce Neuronal Degeneration In Vivo
Source: Stem Cell Reports. 2017 Mar 30;8(4):843–55. doi: 10.1016/j.stemcr.2017.03.003 (PMC5390239; doi:10.1016/j.stemcr.2017.03.003)
Supplement: Document S1. Figures S1–S3 and Table S1 [file mmc1.pdf]

Stem Cell Reports, Volume *8*

Supplemental Information

Sporadic ALS Astrocytes Induce Neuronal Degeneration In Vivo

Kun Qian, Hailong Huang, Andrew Peterson, Baoyang Hu, Nicholas J. Maragakis, Guo-li Ming, Hong Chen, and Su-Chun Zhang

Supplemental Figures and legends

Figure S1. Characteristics of pluripotent stem cells, related to Fig1. (A) A representative phase contrast image of an iPSC colony. Scale bar=50μm. (B-F) The iPSCs were stained for alkaline AKP (C), SOX2 (D), NANOG (E), OCT4 (F) and SSEA4 (G). Scale bar=50μm. (G-I) Representative sections of teratomas formed by the iPSCs showing ectoderm (H), mesoderm (I), and endoderm (J) tissues. Scale bar=50μm. (J) Karyotypes of sALS-1 and sALS-2 iPSCs at passage 40.

Figure S2. Integration of non-ALS and sALS human astrocytes into the adult spinal cord, related the result part '*Neural cells from sALS iPSCs integrate into the adult mouse spinal cord*'. (A) Adjacent sections of the transplanted spinal cord showing distribution and overlap (arrows) of $hGFAP^{+}$ cells (left panel) and hNu^{+} cells (right panel). The boxed area is magnified on the left corner. (B, C) Cross sections showing relationship between $hGFAP^{+}$ human astrocytes and $ChAT^{+}$ MNs or $Map2^{+}$ neurons for non-ALS (B) and ALS (C) groups in the gray matter. (D, E) Immunostaining shows relationship between $hGFAP^{+}$ human astrocyte processes and $laminin^{+}$ blood vessels in non-ALS (D) and sALS (E) groups. Scale bar=50μm.

Figure S3. Ubiquitin inclusions in $ChAT^{+}$ MNs in different segments of the transplanted spinal cord, related to Fig 4B. (A, B) Staining for ubiquitin showing ubiquitin inclusions (arrows) in $ChAT^{+}$ MNs in the spinal cord transplanted with sALS cells (B) and non-ALS cells (A). The transplanted cells were marked by hGFAP. The arrows indicate ubiquitin inclusions on the $ChAT^{+}$ cell body. Scale bar=50μm.

Fig S1

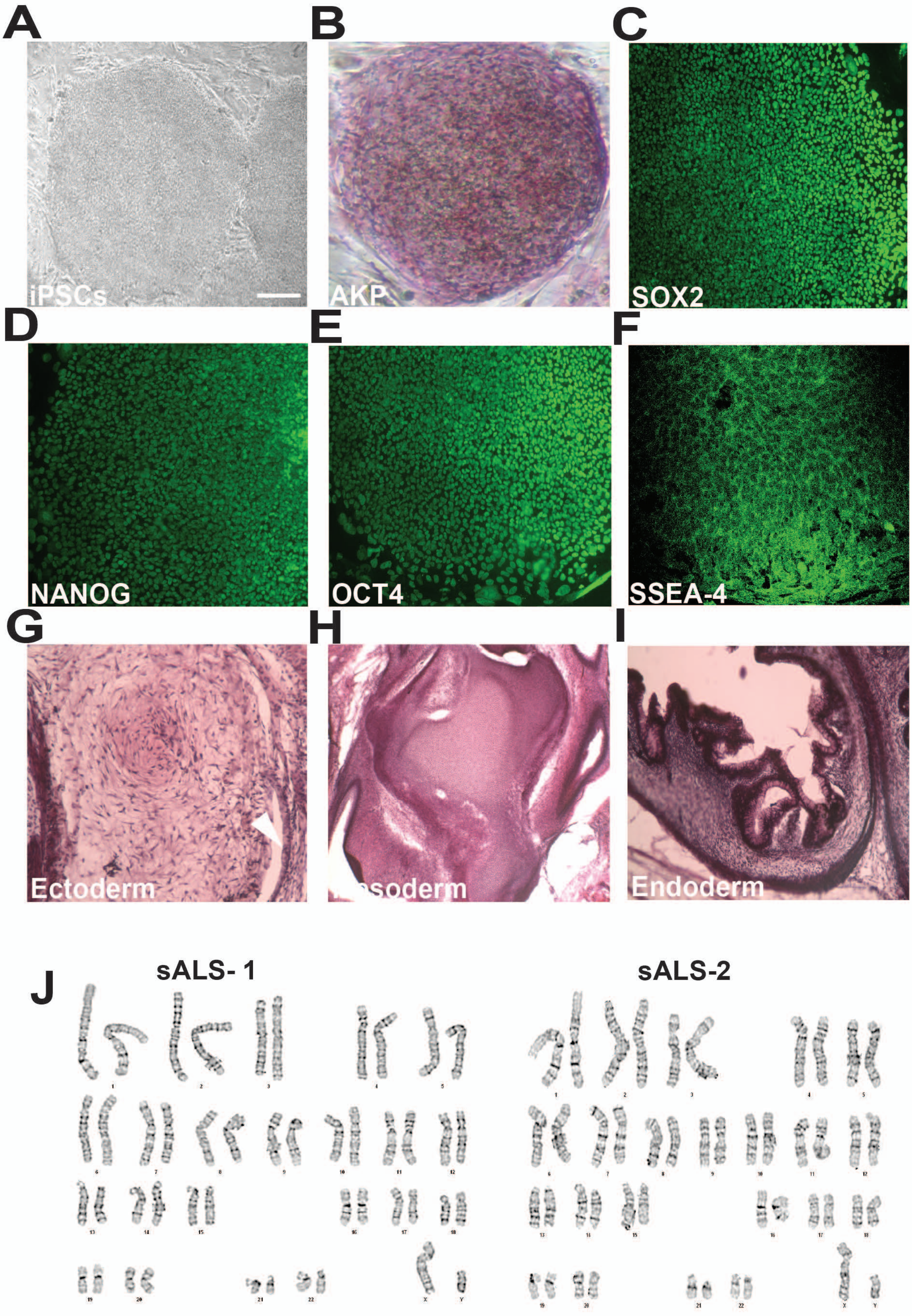
A
B
C
iPSCs
AKP
SOX2
D
E
F
NANOG
OCT4
SSEA-4
G
H
I
Ectoderm
Endoderm
J
sALS- 1
sALS-2

Fig S2

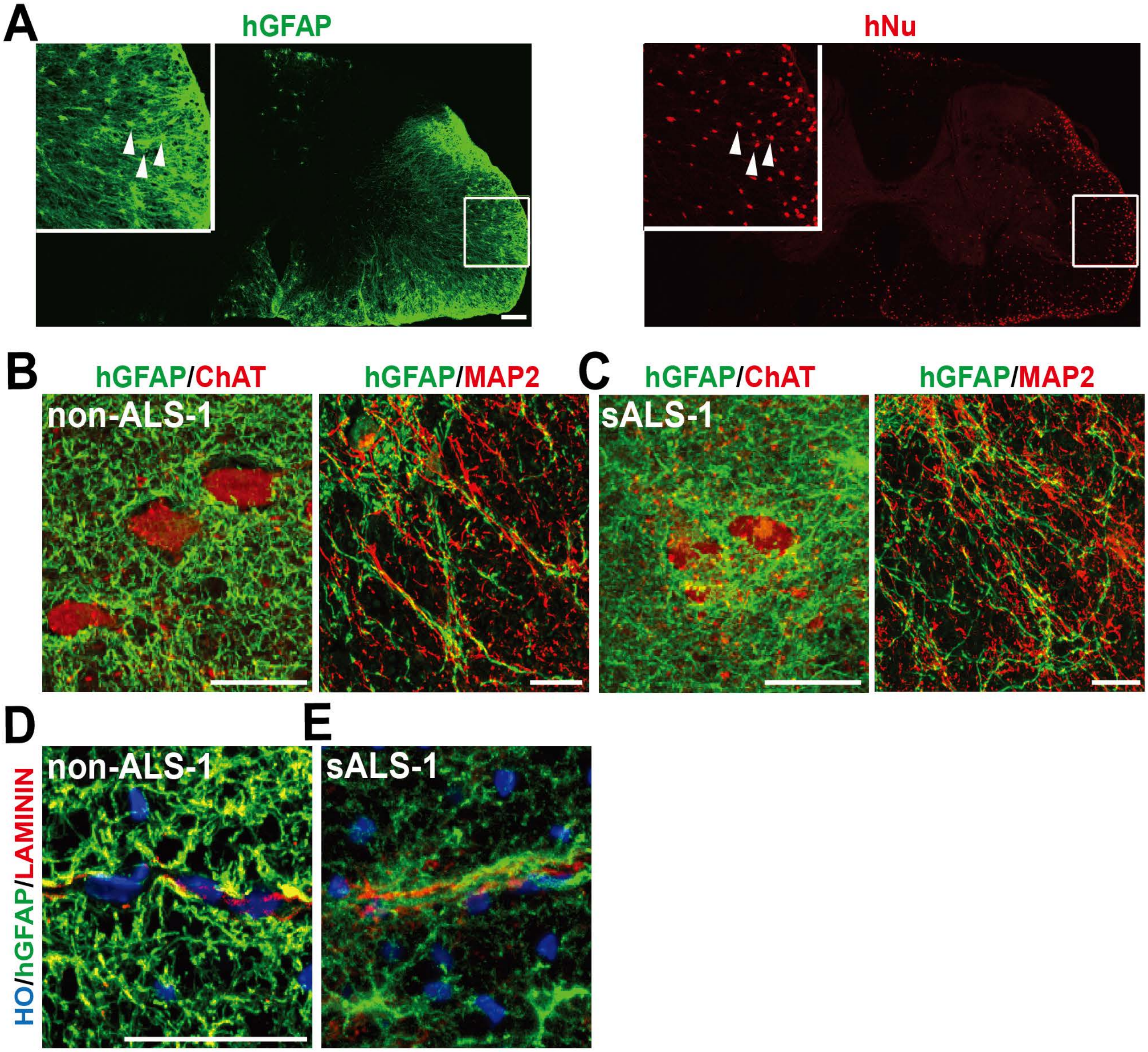
A
hGFAP
hNu
B
hGFAP/ChAT
hGFAP/MAP2
non-ALS-1
C
hGFAP/ChAT
hGFAP/MAP2
sALS-1
D
HO/hGFAP/LAMININ
non-ALS-1
E
sALS-1

Fig S3

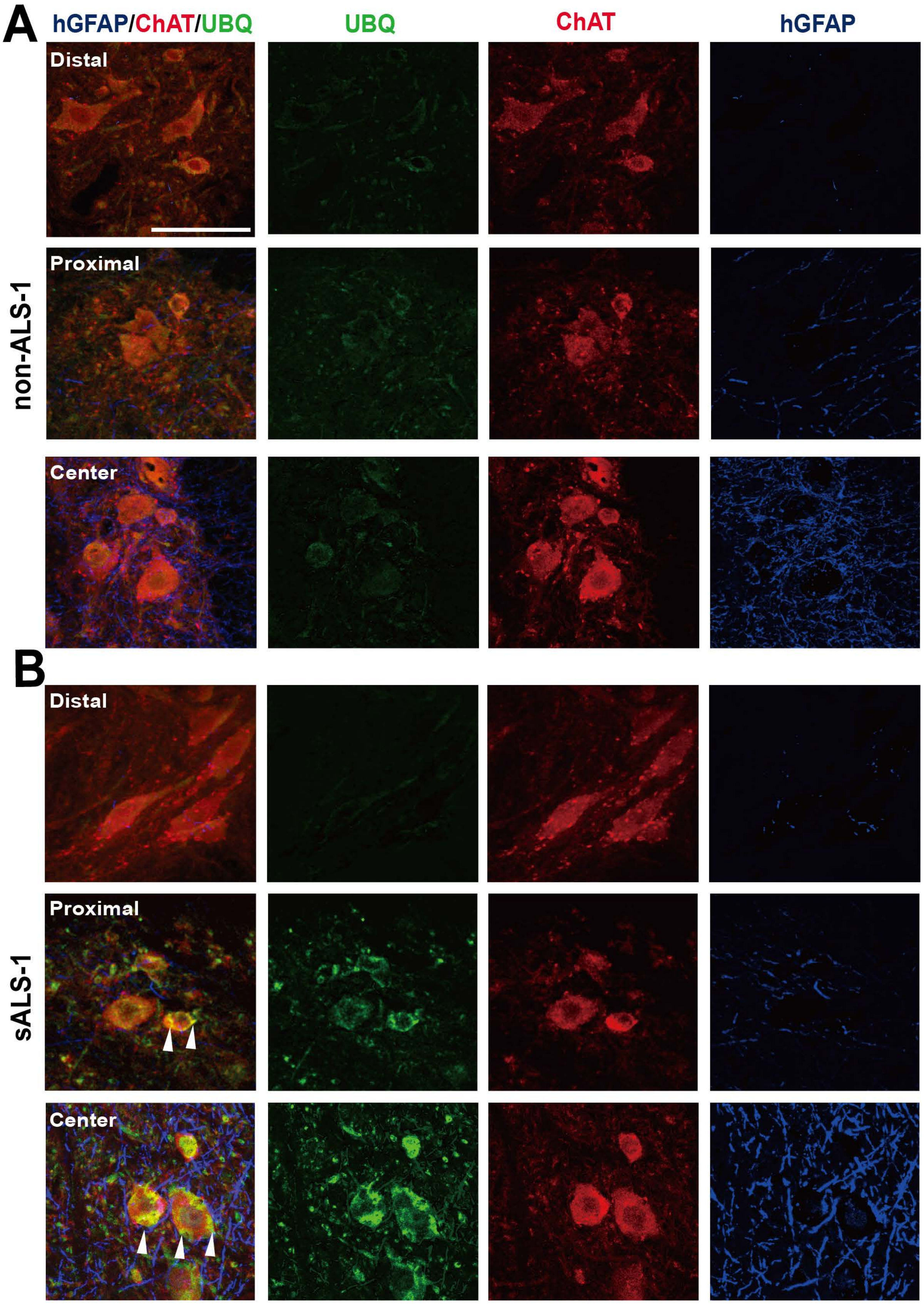
A
hGFAP/ChAT/UBQ
UBQ
ChAT
hGFAP
Distal
Proximal
Center
non-ALS-1
B
Distal
Proximal
Center
sALS-1

Table S1 the list of antibodies used in the study, related to all figures.

Antibody	Isotype	Dilution	Source (Catalog no.)
BTX-488		1:500	Life technologies (B13422)
ChAT	Goat IgG	1:300	Chemicon & Millipore (AB144P)
GFAP	Rabbit IgG	1:5000	DAKO (Z0334)
GAD65	Rabbit IgG	1:2000	Chemicon & Millipore (AB1511)
Human Nuclei	Mouse IgG	1:200	Chemicon & Millipore (MAB1281)
hGFAP	Mouse IgG	1:500	Stem Cells, Inc (AB-123-U-050)
Neurofilament 200	Rabbit IgG	1:1000	Sigma (N4142)
TUBULIN	Rabbit IgG	1:4000	Chemicon & Millipore (AB9354)
MAP2	Rabbit IgG	1:5000	Chemicon & Millipore (AB5622)
NeuN	Rabbit IgG	1:500	Chemicon & Millipore (ABN78)
OLIG2	Rabbit IgG	1:500	Chemicon & Millipore (AB9610)
OTX2	Goat IgG	1:2000	R&D (AF1979)
HOXB4	Rat IgG	1:50	DSHB (I12 anti-Hoxb4)
SYNAPTOPHYSIN	Mouse IgG	1:1000	Chemicon & Millipore (MAB5258)
GLT-1	Guinean pig IgG	1:20000	Chemicon & Millipore (AB1783)
GLYT2	Guinean pig IgG	1:1000	Chemicon & Millipore (AB1773)
UBIQUITIN	Rabbit IgG	1:1000	DakoCytomation (Z0458)
