# Supplementary material for: Sporadic ALS Astrocytes Induce Neuronal Degeneration In Vivo
Source: Stem Cell Reports. 2017 Mar 30;8(4):843–55. doi: 10.1016/j.stemcr.2017.03.003 (PMC5390239; doi:10.1016/j.stemcr.2017.03.003)
Supplement: Document S2. Article plus Supplemental Information [file mmc2.pdf]

Stem Cell Reports
Article
OPEN ACCESS

Sporadic ALS Astrocytes Induce Neuronal Degeneration In Vivo

Kun Qian,[1,7] Hailong Huang,[2,7] Andrew Peterson,[3] Baoyang Hu,[3] Nicholas J. Maragakis,[4] Guo-li Ming,[4,5] Hong Chen,[2,*] and Su-Chun Zhang[3,6,*]
[1]Department of Reproductive Medicine
[2]Department of Rehabilitation Medicine
Tongji Hospital, Tongji Medical College, Huazhong University of Science and Technology, Wuhan 430030, China
[3]Waisman Center, University of Wisconsin, 1500 Highland Avenue, Madison, WI 53705, USA
[4]Department of Neurology
[5]Institute for Cell Engineering
Johns Hopkins University School of Medicine, Baltimore, MD 20036, USA
[6]Departments of Neuroscience and Neurology, University of Wisconsin, Madison, WI 53705, USA
[7]Co-first author
*Correspondence: chenhong1129@hotmail.com (H.C.), suchun.zhang@wisc.edu (S.-C.Z.)
http://dx.doi.org/10.1016/j.stemcr.2017.03.003

SUMMARY

Astrocytes from familial amyotrophic lateral sclerosis (ALS) patients or transgenic mice are toxic specifically to motor neurons (MNs). It is not known if astrocytes from sporadic ALS (sALS) patients cause MN degeneration in vivo and whether the effect is specific to MNs. By transplanting spinal neural progenitors, derived from sALS and healthy induced pluripotent stem cells (iPSCs), into the cervical spinal cord of adult SCID mice for 9 months, we found that differentiated human astrocytes were present in large areas of the spinal cord, replaced endogenous astrocytes, and contacted neurons to a similar extent. Mice with sALS but not non-ALS cells showed reduced non-MNs numbers followed by MNs in the host spinal cord. The surviving MNs showed reduced inputs from inhibitory neurons and exhibited disorganized neurofilaments and aggregated ubiquitin. Correspondingly, mice with sALS but not non-ALS cells showed declined movement deficits. Thus, sALS iPSC-derived astrocytes cause ALS-like degeneration in both MNs and non-MNs.

INTRODUCTION

Amyotrophic lateral sclerosis (ALS) is a late onset neurodegenerative disease characterized by a progressive loss of motor neurons (MNs) in the cerebral cortex, brainstem, and spinal cord. While a small number (5%–10%) of patients are associated with mutations in C9orf72, superoxide dismutase 1 (SOD1), TDP43, FUS, VCP, SQSTM1, OPTN, and TBK1 (Cirulli et al., 2015; Maruyama et al., 2010), the vast majority (90%) do not have an obvious family history (Gros-Louis et al., 2006), referring to as sporadic ALS (sALS). The cause of ALS remains largely unknown.

Although ALS is primarily an MN disease, non-neuronal cells have been shown to play an important role in its pathogenesis. Embryonic integration of healthy glial cells in SOD1 (G37R and G85R) transgenic mice mitigated or delayed the disease process with an average lifespan extension of 1.6 months (Clement et al., 2003), demonstrating the involvement of glia in disease progression. Similarly, knock down of mutant SOD1 (G37R or G85R) in astrocytes of transgenic mice through crossing of G37R (or G85R)^{flox} mice with Cre mice driven by the glial fibrillary acidic protein (GFAP) transcription control element delayed disease progression by 60 days and prolonged survival by 48 days (Wang et al., 2011; Yamanaka et al., 2008). The role of mutant protein-expressing astrocytes in ALS pathogenesis is further demonstrated by compromised survival of mouse or human embryonic stem cell-derived MNs when co-cultured with astrocytes that are isolated from SOD1^{G93A} transgenic mice (Di Giorgio et al., 2007; Nagai et al., 2007) or those expressing SOD1^{G37R} protein (Marchetto et al., 2008). We have recently shown that ALS (SOD1^{D90A}) patient-induced pluripotent stem cell (iPSC)-derived neural progenitors, following transplantation into the spinal cord of severe combined immunodeficiency (SCID) mice and differentiation to astrocytes, impair the survival of MNs (Chen et al., 2015). Thus, astrocytes expressing ALS-associated proteins indeed impair MN survival and potentiate the disease progression.

The role of sALS astrocytes on MN is controversial. Astrocytes, derived from postmortem spinal cord tissues of sALS patients, selectively impaired the survival of MNs after 120 hr of co-culture (Haidet-Phillips et al., 2011), possibly by the caspase-independent necroptosis pathway (Re et al., 2014). Nevertheless, such an effect may be due to the reactive astrocytes that were cultured from the postmortem ALS patient spinal cord. To address this issue, Kaspar and colleagues generated induced neural progenitor cells from sALS patient fibroblasts and differentiated the progenitors into astrocytes (i-astrocytes) before co-culturing with MNs. Under this condition, the sALS i-astrocytes impaired the survival of MNs (Meyer et al., 2014). However, astrocytes from ALS patient iPSCs had no obvious toxicity to MNs in culture (Re et al., 2014; Serio et al., 2013). The contrasting results suggest that the effects of astrocytes on MNs may be influenced by culture conditions, raising

This is an open access article under the CC BY license (http://creativecommons.org/licenses/by/4.0/).

the question of whether sALS astrocytes play a role in MN degeneration, especially in vivo. It is also mysterious why ALS astrocytes impair MNs specifically. To address these questions, we have established a chimeric mouse model in which neural progenitors from sALS patient iPSCs differentiate to astrocytes and replace their counterparts in the SCID mouse spinal cord over a 9-month period. Under this condition, MNs adjacent to the sALS astrocytes exhibit signs of degeneration with concomitant mouse behavioral deficits. Interestingly, non-MNs are also lost, even at an earlier time.

RESULTS

Neural Cells from sALS iPSCs Integrate into the Adult Mouse Spinal Cord

To assess the role of sALS astrocytes in vivo, we first generated iPSC lines, sALS-1 (JH026) and sALS-2 (JH028), from fibroblasts of 54- and 68-year-old patients (Johns Hopkins IRBNA_00021979), respectively, by the non-integrating Sendai virus (Ban et al., 2011). These two patients were diagnosed with sALS as they did not have a family history and lack of mutations in the C9orf72 gene, the most commonly missed gene in the diagnosis of sALS. Both patients presented symptoms relating to MN degeneration of the spinal cord but not brainstem or cortex. One patient survived for 5 years from diagnosis, whereas the other survived for 12 years and was regarded as slow progressing type. The iPSCs exhibited characteristic stem cell morphology (Figure S1A), expressed pluripotency markers, including alkaline phosphatase, SOX2, NANOG, OCT4, and SSEA4 (Figures S1B–S1F), and formed teratomas in vivo (Figures S1G–S1I). They exhibited normal karyotypes when assayed at passage 40 (Figure S1J).

We then generated spinal progenitors from the two sALS iPSC lines and two non-ALS (control) PSC lines (IMR90 iPSCs and H9-GFP embryonic stem cells [ESCs]) according to our established method (Li et al., 2005). The vast majority (80%) of the cells differentiated for 35–42 days expressed ventral spinal cord markers OLIG2 and HOXB4, but not OTX2, an anterior marker (Figures 1A and 1B). We then transplanted the progenitors into SCID mice in the ventral spinal cord at the cervical enlargement (C5-C6) level. Nine months later, both ALS and non-ALS cells, identified by human nuclei (hNu) staining, were present in large areas of the spinal cord, spreading 11 ± 1 mm in length longitudinally (Figure 1C). Stereological quantification of hNu^+ cells indicated that $7.9 \pm 0.2 \times 10^5$ cells were present in the spinal cord receiving ALS cell transplant and $9.4 \pm 1 \times 10^5$ cells in the cord receiving the non-ALS cell graft (Figure 1D). Thus, both ALS and non-ALS neural cells survived and migrated in the adult mouse spinal cord. Analysis of cell identity by immunocytochemistry indicated that 83.9% ± 3.1% of the hNu^+ cells were positive for GFAP (Figure 1E), 10.4% ± 2.5% of the hNu^+ cells were $NeuN^+$ (but not $ChAT^+$), and 6.7% ± 0.1% positive for NG2, suggesting that the grafted neural progenitors predominantly become astrocytes. In the transplant site, 90% of the $GFAP^+$ cells were positive for hGFAP (Figures 1F and 1G). Indeed, the vast majority of hNu^+ cells also exhibited $hGFAP^+$ cells (Figure S2A). Thus, the majority astrocytes were of human origin near the transplant site.

Morphologically, both ALS and non-ALS human astrocytes were larger with more processes than their mouse counterparts. Like endogenous astrocytes, the grafted astrocytes and their processes closely opposed host neurons, including MNs and axons (Figures S2B and S2C). Human astrocytes projected to blood vessels with their endfeet closely surrounding the blood vessel (Figures S2D and S2E). Thus, the transplanted astrocytes, both ALS and non-ALS, morphologically integrate into the mouse spinal cord.

ALS Astrocytes Exhibit Reactive Properties

In the transplant-created chimeric model, neural progenitors were transplanted into one site. The grafted progenitors migrate away and gradually differentiate to astrocytes (Chen et al., 2015). Hence, the closer to the graft site, the earlier and more complete replacement by the human astrocytes. The more distal areas from the graft site are occupied by the astrocytes that are recently differentiated from newly migrated progenitors. Such a gradient of cell replacement allows a "dynamic" assessment of relative effects of human astrocytes on host cells/tissues in a single spinal cord. We arbitrarily divided the grafted spinal cord into three domains. The center domain is the area surrounding the graft site in which over 50% of the cross-section is occupied by human ($hGFAP^+$) astrocytes, representing the longest interaction between human cells and host tissues. The distal domain has <20% areas occupied by recently arrived human astrocytes and the proximal domain lies between this, having a 20%–50% area with the presence of human astrocytes (Figure 2A).

Astrogliosis is common in ALS patients (Schiffer et al., 1996; Yu et al., 2001) and transgenic mice (Bruijn et al., 1997; Hall et al., 1998; Howland et al., 2002; Wong et al., 1995), although the time course of astrogliosis, relative to MN degeneration and the onset of symptoms, seems to vary. By measuring the immunofluorescent intensity, we found that the relative intensity for human-specific GFAP in the center of ALS cell-transplanted mouse spinal cord was significantly higher than that in the non-ALS cell-transplanted groups (Figure 2D). At a higher magnification, ALS astrocytes showed hypertrophy

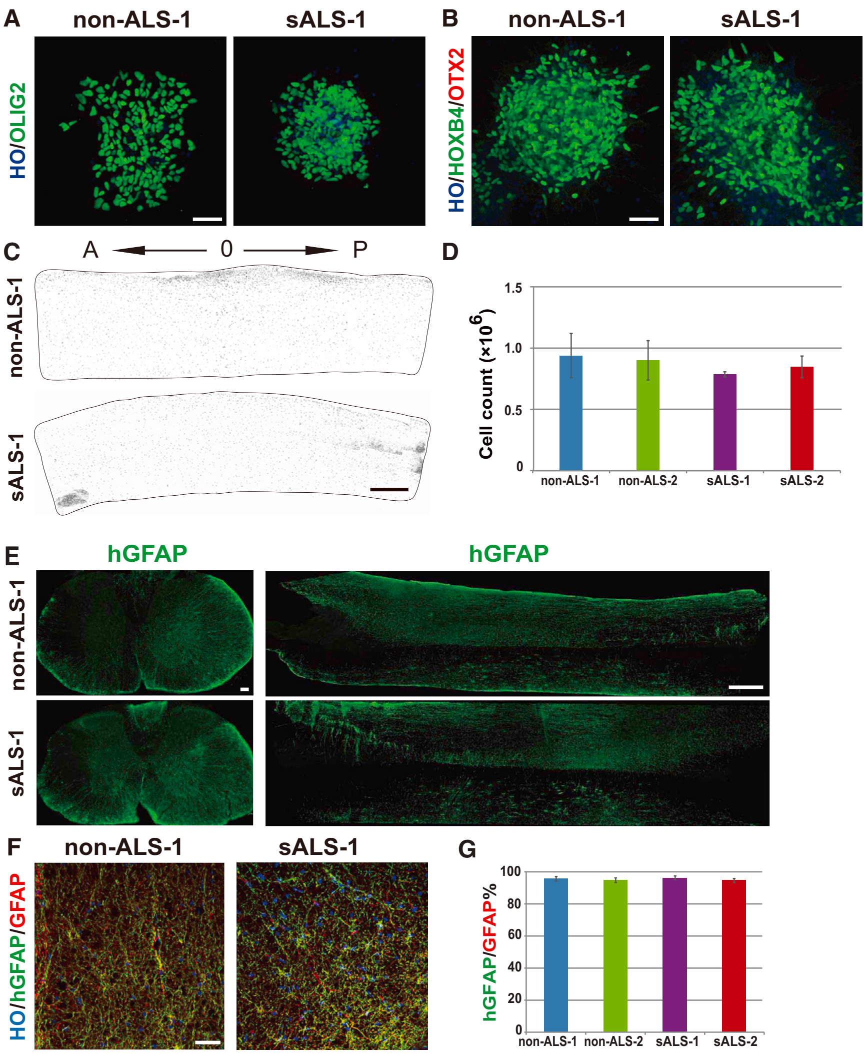

Figure 1. Characterization of Transplanted Human Cells in the Adult Mouse Spinal Cord

(A) Immunofluorescent image of OLIG2 expression in differentiated neural progenitors at day 35. Scale bar, 50 μm. From three independent experiments.

(B) Immunofluorescent image of HOXB4/OTX2 expression in differentiated neural progenitors at day 35. Scale bar, 50 μm. From three independent experiments.

(C) Sagittal sections show the presence of sALS and non-ALS human cells (hNu^+) in the adult spinal cord of SCID mice along the rostral (A) and caudal (P) direction, with "0" indicating the injection site. Scale bar, 500 μm.

(D) Stereological quantification of human cells (hNu^+) in the spinal cord of 10 mm in length covering the transplant site. Data are represented as mean ± SEM from three independent experiments, with n = 6 mice for each group.

(E) The coronal and longitudinal distribution of hGFAP-expressing astrocytes, Scale bar, 50 μm (left panel), Scale bar, 1000 μm (right panel).

(F) Representative transplanted spinal cord sections stained for human-specific GFAP (hGFAP, green) and pan-GFAP (red), Scale bar, 50 μm.

(G) Quantification of $hGFAP^+$ and $GFAP^+$ cells in the transplanted spinal cord. Data are represented as mean ± SEM with n = 6 mice for each group.

See also Figures S1 and S2.

of cellular processes (Figures 2B and 2C). In the distal domain (<20%) the astrocytes were smaller and had longer and thinner processes than those in the center and proximal domains (Figures 2B and 2C). In this distal domain there was no obvious difference in hGFAP intensity between sALS and the non-ALS groups (Figure 2D).

One of the astrocyte functions is to remove glutamate released in the synaptic cleft through sodium-dependent excitatory amino acid transporters EAAT2 (GLT1), and reactive astrocytes often display a decreased level of GLT1 (Bruijn et al., 1997). Densitometry indicated that the relative intensity of GLT1 in sALS astrocytes was significantly lower (70%) than that of non-ALS astrocytes (Figures 2E–2G) in the center domain, while in the distal domain the relative intensity of GLT1 had no difference between the groups (Figures 2E–2G). Thus, sALS astrocytes show reactive characteristics.

Non-MNs Are Lost Earlier than MNs in sALS Cell-Transplanted Mice

In culture, ALS astrocytes specifically impair MN survival (Di Giorgio et al., 2007, 2008; Nagai et al., 2007). To determine if sALS astrocytes similarly cause MN loss specifically in vivo, we quantified the number of $ChAT^+$ MNs and $ChAT^-/NeuN^+$ non-MNs in the transplanted cord (Figure 3). In the transplanted site (center domain), we found that the number of MNs was reduced by about 40% and non-MNs reduced by 27%, respectively (Figure 3). This result indicates that sALS astrocytes indeed impair neurons but that the effect is not specific to MNs.

In our chimeric model, transplanted cells migrate along the spinal cord, differentiate to astrocytes, and integrate into the host tissue over time. When quantified in different domains of the transplanted cord, we found

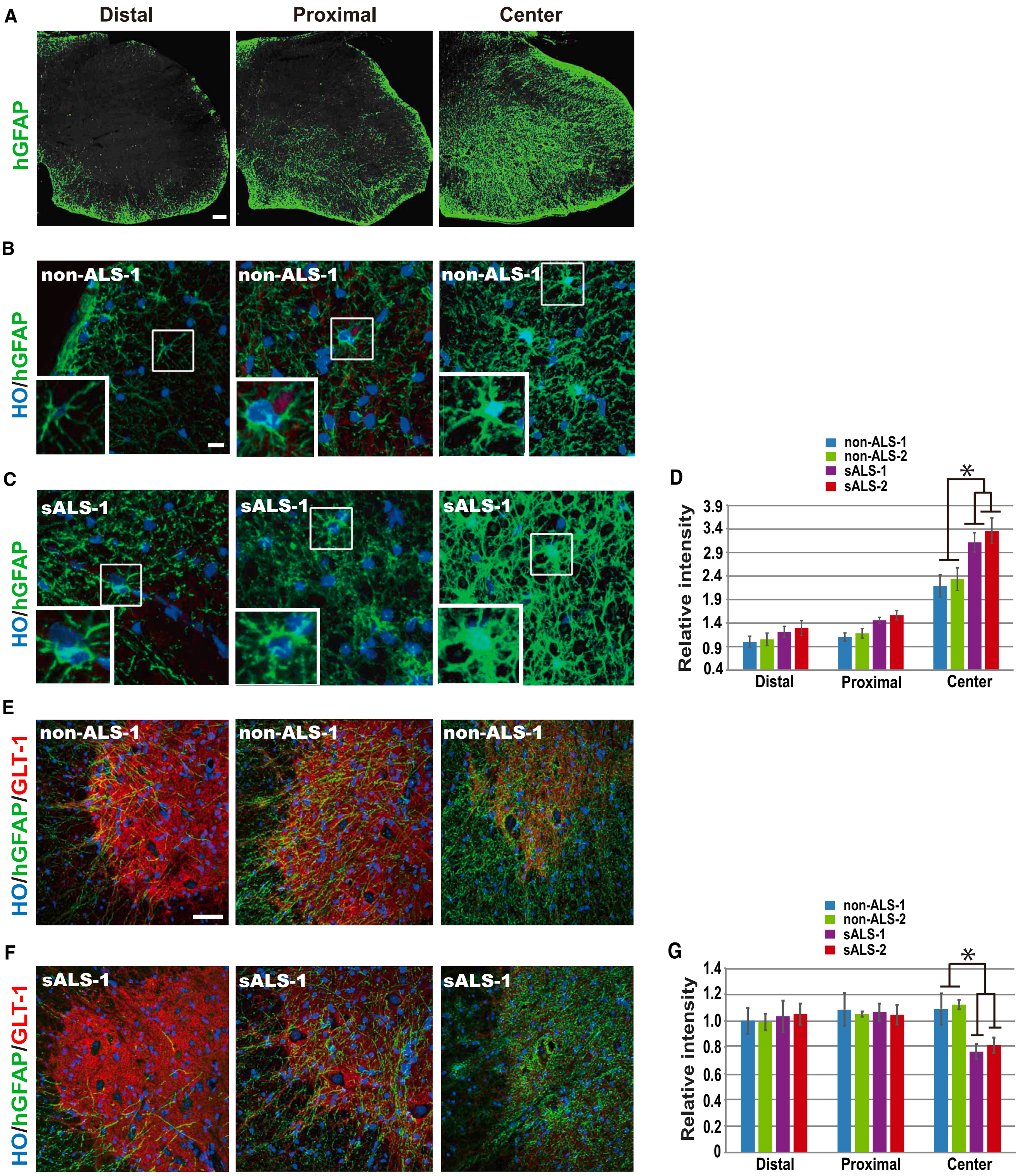

Figure 2. Characterization of Transplanted Human Astrocytes

(A) Representative sections with presence of hGFAP^{+} cells (green) in the distal (<20%), proximal (20%–50%), and center (>50%) areas of the transplanted spinal cord. Scale bar, 50 μm.

(legend continued on next page)

that MN number was reduced in the center domain but not in the distal and proximal domains of the spinal cord transplanted with sALS cells compared with that with non-ALS cells (Figures 3A, 3B, and 3E). Non-MNs were reduced by around 27% in the proximal and the center domains but not the distal domain of the spinal cord receiving sALS cell transplantation (Figures 3C, 3D, and 3F). Since the area with fewer astrocytes is distant from the transplant site and is integrated by grafted cells later, the loss of non-MNs but not MNs in the proximal domain suggests that non-MNs are lost earlier than MNs in sALS cell-transplanted animals.

sALS Astrocyte-Transplanted Cord Displays ALS-like Neuronal Pathology

MNs in ALS patients (Carpenter, 1968; Hirano et al., 1984) as well as those cultured from ALS patient iPSCs (Chen et al., 2014) exhibit aggregation of proteins, especially neurofilaments. To discern if sALS astrocytes result in a general bystander effect on neurons or induce an ALS-like pathological change in host MNs, we stained the transplanted cord for neurofilament and ubiquitin. Interestingly, neurofilament, weakly present in the cell body and proximal end of axons of $ChAT^+$ MNs in contact with non-ALS astrocytes, exhibited irregular shapes in the cell body and major processes of the $ChAT^+$ MNs that were in contact with sALS astrocytes (Figure 4A). The presence of neurofilament aggregates in MN cell body and major processes was confirmed by z-section of confocal imagining (Figure 4A). In addition, we observed an increased level of ubiquitin fluorescein in MNs that were in contact with sALS astrocytes. Furthermore, the distribution of ubiquitin was not even, with granular staining surrounding the nucleus and around the cell body (Figures 4B and S3). Almost all the MNs around the transplant site had ubiquitin inclusions, whereas fewer MNs had the ubiquitin aggregates in distal areas (Figures 4B, 4C, and S3). These results suggest that MNs exhibit a pathological change like that seen in ALS patients.

Besides MNs, other neuronal types, especially inhibitory neurons, have also exhibited changes in ALS (Martin and Chang, 2012; Martin et al., 2007). Our present observation indicates that non-MNs may undergo degeneration earlier than MNs (Figure 3). One hypothesis is that reduction in inhibitory inputs to MNs results in hyper-excitability and ultimately degeneration of MNs (Wainger et al., 2014). To discern if there is an alteration in inhibitory inputs, we quantified glycinergic and GABAergic terminals on MNs in the transplanted mouse spinal cord. We found that GAD65 punctae on the MN body were decreased by more than 50% (Figures 4D and 4E) and the glycine transporter 2 (GLYT2) punctae by about 20% (Figures 4F and 4G) compared with sALS with non-ALS groups. This result suggests a potential link between loss of inhibitory neurons and MN degeneration.

Mice Transplanted with sALS Cells Exhibit Pre-synaptic Defects at NMJ

The loss of MNs in the spinal cord suggests denervation of muscles in the transplanted mice. Since MN loss occurs mainly in the cervical enlargement area, we examined the neuromuscular junctions (NMJs) in the shoulder-deltoid muscles. Pre-synaptic nerve terminals were detected with a synaptophysin antibody and postsynaptic endplates with α-bungarotoxin (BTX). Since cells were transplanted to only one side from which cells migrate to the contralateral side, we compared muscle denervation between transplant and non-transplant sides as well as between the spinal cords with ALS and non-ALS cell transplants. The number of BTX-positive postsynaptic endplates was significantly decreased in the transplant side compared with the untransplanted side of the spinal cord with an sALS cell graft or both sides with non-ALS cell transplants (Figures 5A–5C). In the untransplanted side of the sALS group, the number of BTX-positive postsynaptic endplates was lower than that in the control groups but the difference was not statistically significant (Figure 5C). NMJs that had both red (synaptophysin) and green (BTX) staining were considered as innervated, while NMJs stained with only BTX were considered denervated. The percentage of denervation in the sALS cell transplant group was 57.7%, significantly higher than the non-ALS cell transplant groups (Figures 5A, 5B, and

(B and C) Human astrocytes ($hGFAP^+$) from non-ALS (B) and sALS (C) iPSCs in the spinal cord with human cells present in the distal, proximal, and center areas. The boxed area is magnified at the bottom left corner. Scale bar, 10 μm.

(D) Relative fluorescent intensity of human-specific GFAP (mean ± SEM) in cross-sections of the spinal cord with human cells present in the distal, proximal, and center areas. Significance was assessed using one-way ANOVA followed by Tukey's multiple comparison tests (n = 6 mice per group, *$p < 0.05$).

(E and F) GLT1 staining (red) in human ($hGFAP^+$, green) astrocytes from non-ALS (E) and sALS (F) in the spinal cord with human cells present in the distal, proximal, and center areas. Scale bar, 50 μm.

(G) Relative intensity of GLT-1 (mean ± SEM) in cross-sections of the spinal cord with human cells present in the distal, proximal, and center areas. Significance was assessed using one-way ANOVA followed by Tukey's multiple comparison tests (n = 6 mice per group, *$p < 0.05$).

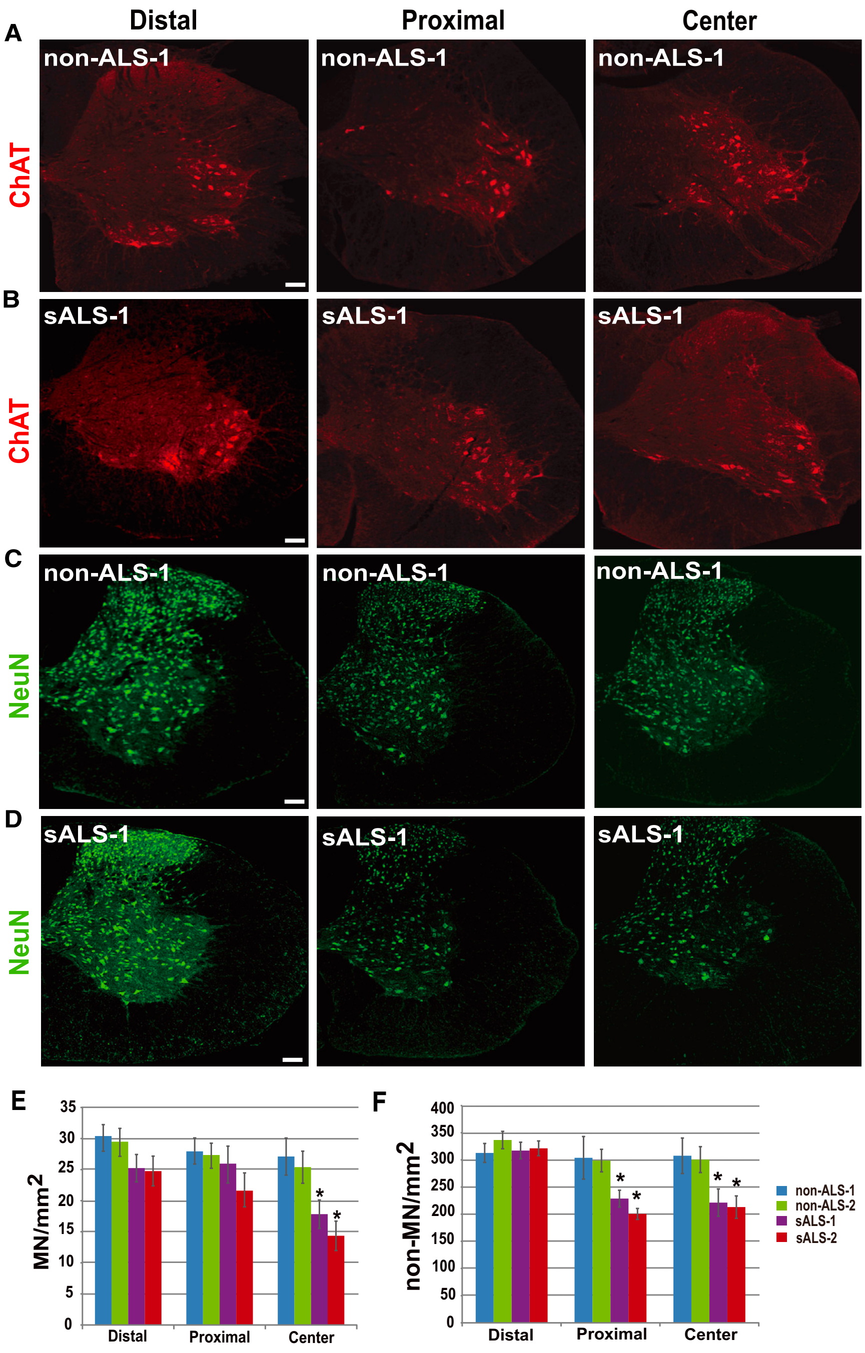

Figure 3. Characterization of MNs and Non-MNs in the Transplanted Spinal Cord

(A and B) ChAT staining (red) shows MNs in sections with different proportions of human astrocyte incorporation.

(C and D) NeuN staining (green) shows neurons in sections with different proportions of human astrocyte incorporation.

(E and F) Quantification of MNs (E) and non-MNs (F) in the spinal cord with human cells present in the distal, proximal, and center areas. Data are represented as mean ± SEM. Significance was assessed using one-way ANOVA followed by Tukey's multiple comparison tests (n = 6 mice per group, $*p < 0.05$).

Scale bar, 50 μm.

5D). These results suggest pre-synaptic defects at the NMJ in the mice transplanted with sALS cells.

Mice with sALS Cell Transplant Exhibit Motor Deficits

Analysis of locomotor behaviors of mouse forelimbs before and 3, 6, and 9 months after transplantation (Figure 6A) showed that the grip strength of the right forelimb decreased at 6 and 9 months post transplantation with sALS cells. The animals receiving the non-ALS cell transplant showed no obvious changes (Figure 6B). Treadscan analysis, which detects fine gait changes, showed decreased stride length, average print area, and maximum lateral deviation (the closest distance from right foot to the body axis) in animals at 9 months post transplantation with ALS cells compared with those with non-ALS cell transplant (Figures 6C–6E). These data indicate that the

proximal muscles of right forelimb of the ALS cell-transplanted animals are weak.

DISCUSSION

The transplant-created chimera enables us to investigate the effects of human neural cells in the adult mouse spinal cord (Chen et al., 2015). Using this model, we have shown that neural cells from sALS patient iPSCs, especially astrocytes, integrate into the mouse spinal cord to a similar degree as healthy cells. It is the sALS cells that induce degenerative changes in both MNs and non-MNs of the host, which corresponds to the mouse motor behavioral deficits. By taking advantage of the time-dependent integration of human cells in this model, we have discovered that non-MNs are lost earlier than MNs, with a corresponding reduction of inhibitory nerve terminals in MNs. Thus, the effect of sALS astrocytes on neural degeneration is not specific to MNs, and non-MNs may mediate MN degeneration.

Involvement of glial cells in the pathogenesis of ALS has been suggested from a series of studies where disease-causing proteins are specifically expressed in glial cells in transgenic animals (Yamanaka et al., 2008) and when MNs are cultured with glial cells that express mutant ALS proteins (Di Giorgio et al., 2007, 2008; Nagai et al., 2007). The involvement of glia in sALS is suggested by recent observations that astrocytes, derived from neural progenitor cells that were reprogrammed from sALS patient fibroblasts (i-astrocytes), impaired the survival of MNs (Meyer et al., 2014). Nevertheless, astrocytes generated in a similar manner (via iPSCs) had no obvious effects (Re et al., 2014; Serio et al., 2013), casting a possibility that the in vitro toxic effects of astrocytes may be influenced by culture conditions. Our present study offers in vivo evidence that non-MN cells, especially astrocytes, may participate in the neural degeneration in sALS. This is demonstrated by the fact that neural progenitors differentiated from sALS patient but not healthy PSCs, generate non-MN cells following transplantation into the spinal cord of SCID mice, and cause neuronal degeneration and corresponding motor deficits in mice. It should be noted that one of the control transplants was performed at a different time yet the result was very similar, highlighting the consistency of the effect of the astrocytes and the reproducibility of the experiments. Furthermore, we show here that cells from both sALS patients have similar effects. ALS is heterogeneous. Although we excluded the possibility of C9orf72 mutations, the main contributor of genetic components to sALS, we cannot rule out other albeit extremely rare mutations and those that have not yet been discovered. Since the majority of the differentiated cells are astrocytes, astrocytes may play important roles in neuronal degeneration. In addition, the neuronal degeneration occurs mostly in the transplant center or near the center but not the distal area. Astrocytes in and around the transplant center are more mature than those in the distal area. This suggests that mature but not immature astrocytes exert toxic effects. This may in part explain why astrocytes generated from ALS patient iPSCs have limited effects (Re et al., 2014; Serio et al., 2013). Astrocytes generated from human ESCs or iPSCs usually exhibit immature phenotypes (Krencik et al., 2011).

Co-culture studies have suggested a specific effect on MNs by ALS astrocytes (Di Giorgio et al., 2007, 2008; Nagai et al., 2007). The reason for the specific effect of ALS astrocytes on MNs remains a mystery. One possibility is that MNs are particularly vulnerable to toxicity, especially in the cell culture environment. In some cases, such a phenomenon may be misinterpreted due to the fact that changes in MN but not non-MN numbers are readily discerned because MNs are usually the minority in the culture system. Because of this mystery, we have paid a particular attention to the effect on MNs and non-MNs. Contrary to the in vitro observations, we found that both MNs and non-MNs degenerate in the spinal cord transplanted with sALS cells. This result suggests that the effect of astrocytes is not specific to MNs. An attempt to identify MN-specific astrocytic factors may be elusive.

What is more intriguing is that non-MNs undergo degeneration earlier than MNs. This is demonstrated by the fact that loss of non-MNs is already obvious in the spinal cord segment that is distant from the injection site and has partial (ventral gray matter) integration by human astrocytes. Unlike the human-mouse chimera created by transplantation of human glial progenitors into the neonatal mouse brain, which results in extensive glial replacement (Windrem et al., 2008), our chimeric model is produced by transplanting human progenitors into one site of the adult mouse spinal cord. The grafted cells migrate along the spinal cord and gradually differentiate to astrocytes over the 9-month period, thus creating a gradient glial replacement, with more complete replacement in the injection site and partial incorporation in distal areas. The more distal areas are those newly populated by grafted human cells. Hence, the loss of non-MNs but not MNs in the segment that has 20%–50% astrocyte incorporation suggests degeneration of non-MNs at an earlier time than MNs. This phenomenon suggests that non-MNs may be at least similarly, if not more, sensitive to ALS astrocytes in vivo. The loss of non-MNs is also observed in the ALS patient spinal cord where glycine binding sites (Hayashi et al., 1981; Whitehouse et al., 1983) and

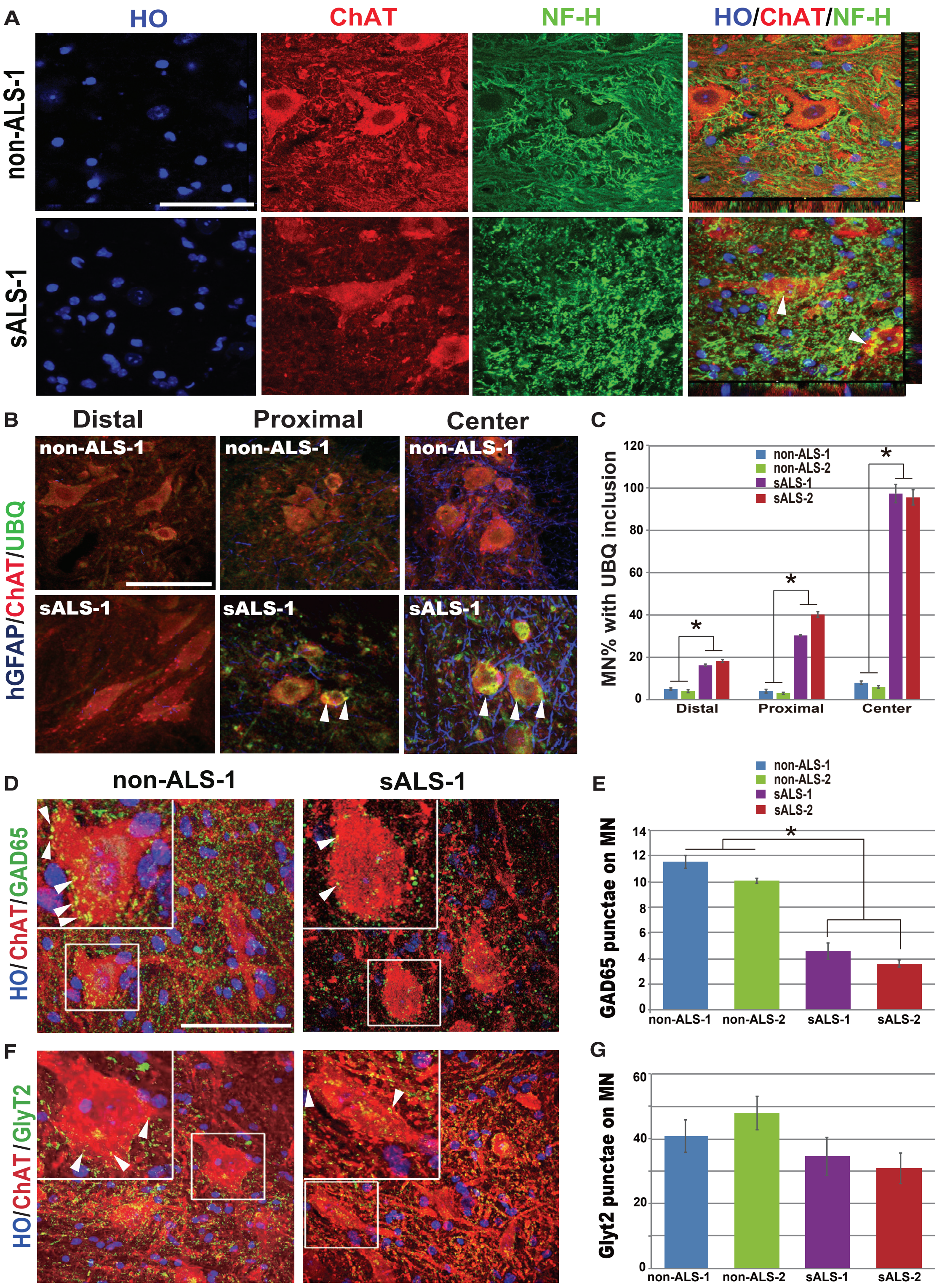

Figure 4. MN Pathology in Transplanted Mice

(A) Staining for NF-H showing NF inclusions (green) in ChAT^{+} MNs in the spinal cord transplanted with sALS cells (lower row) but not in animals transplanted with non-ALS cells (upper row). The arrows indicate neurofilaments aggregation on the ChAT^{+} cell body. Scale bar, 50 μm.

(legend continued on next page)

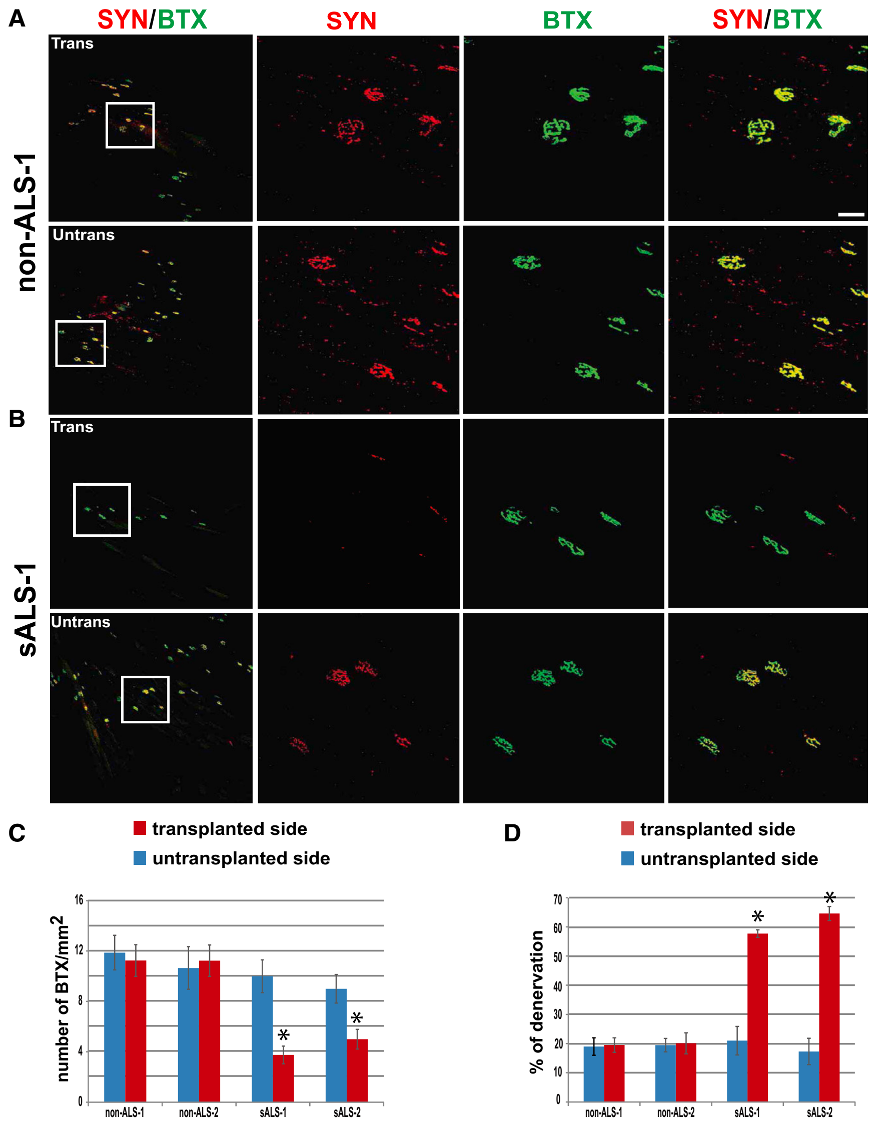

Figure 5. NMJ in Transplanted Mice

(A and B) Representative images of NMJ in deltoid muscle stained for synaptophysin (red) and bungarotoxin (green) in animals transplanted with non-ALS cells (A) and sALS cells (B). Upper row is from the transplant side and the lower row from untransplant side. The inset in the first column is magnified on the right with separate fluorescent channels. Scale bar, 50 μm.

(C) Quantification of BTX^+/SYN^+ NMJ numbers (mean ± SEM). Significance was assessed using one-way ANOVA followed by Tukey's multiple comparison tests (n = 6 mice per group, *$p < 0.05$).

(D) The percentage of denervated (BTX^+/SYN^-) NMJ (mean ± SEM). Significance was assessed using one-way ANOVA followed by Tukey's multiple comparison tests (n = 6 mice per group, *$p < 0.05$).

GABAA receptor subunit mRNA (Petri et al., 2003) are decreased, although it is difficult to track if loss of interneurons occurred early using patient samples. Interestingly, in G93A-hSOD1 mice, $ubiquitin^+$ neurite abnormalities were found in the neuropil of the ventral horn, while MN cell bodies did not display ubiquitin-containing inclusions at pre-symptomatic stages of the disease. In early symptomatic mice, ubiquitin accumulated in smaller neurons with

(B) Ubiquitin staining showing ubiquitin inclusions in $ChAT^+$ MNs in different segments of the spinal cord transplanted with sALS (lower row) or non-ALS (upper row) cells. The arrows indicate ubiquitin inclusions on the $ChAT^+$ cell body. Scale bar, 50 μm.

(C) Quantification of MNs (mean ± SEM) with ubiquitin inclusions in different segments of the spinal cord with sALS or non-ALS human cells. Significance was assessed using one-way ANOVA followed by Tukey's multiple comparison tests (n = 6 mice per group, *$p < 0.05$).

(D and E) Immunostaining and quantification of GAD65 punctae on $ChAT^+$ MNs in the transplanted spinal cord. The boxed area is magnified on the left corner. The arrows indicate GAD65 punctae on the $ChAT^+$ cell body. Data are represented as mean ± SEM. Significance was assessed using one-way ANOVA followed by Tukey's multiple comparison tests (n = 6 mice per group, *$p < 0.05$). Scale bar, 50 μm.

(F and G) Immunostaining and quantification of GLYT2 punctae on $ChAT^+$ MNs in the transplanted spinal cord. The boxed area is magnified on the left corner. The arrows indicate GLYT2 punctae on the $ChAT^+$ cell body. Data are represented as mean ± SEM. Significance was assessed using one-way ANOVA followed by Tukey's multiple comparison tests (n = 6 mice per group). Scale bar, 50 μm.

See also Figure S3

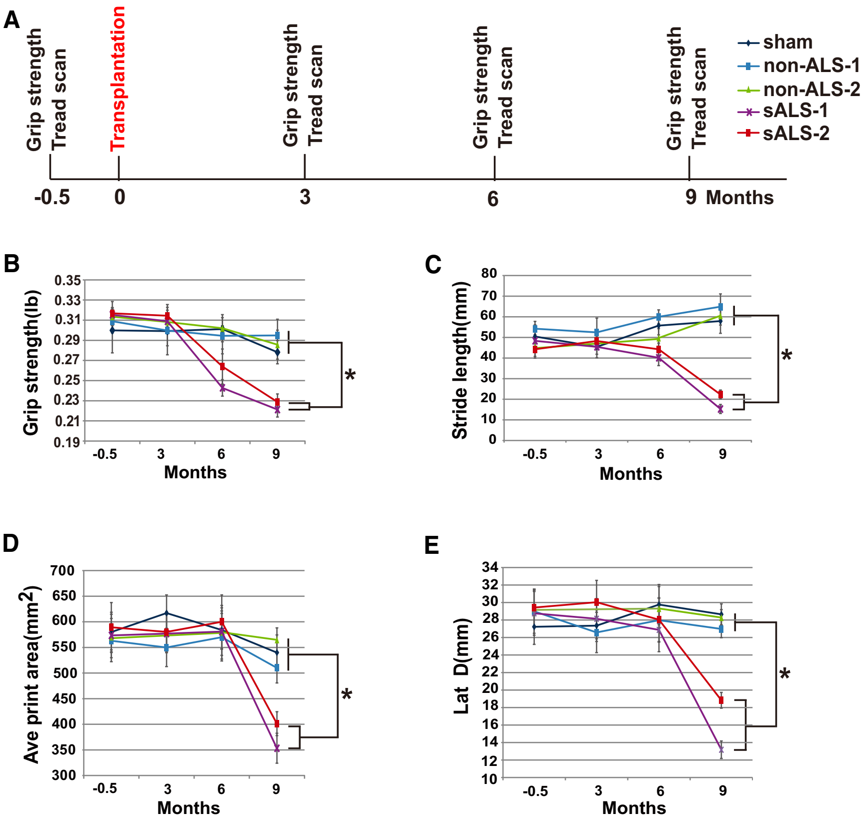

Figure 6. Motor Behavioral Changes in Transplanted Mice

(A) Schematic diagram showing schedules of transplantation and behavior tests.

(B) Change in grip strength (mean ± SEM) in mice receiving non-ALS (1 and 2) and sALS (1 and 2) cell transplantation as well as sham control. Significance was assessed by one-way ANOVA followed by Tukey's multiple comparison tests (non-ALS n = 12 mice, sALS n = 12 mice, sham n = 9 mice, $*p < 0.05$).

(C–E) Treadscan analysis shows changes in stride length (C), average print area (D), decreased maximum lateral deviation (E) in mice receiving non-ALS (1 and 2) and sALS (1 and 2) cell transplantation as well as sham control. Data are represented as mean ± SEM. Significance was assessed by one-way ANOVA followed by Tukey's multiple comparison tests (non-ALS n = 12 mice, sALS n = 12 mice, sham n = 9 mice, $*p < 0.05$).

a fusiform or polygonal morphology but not yet in MN cell bodies (Martin and Chang, 2012; Martin et al., 2007), suggesting degeneration of non-MNs before MNs. The reason why interneurons (non-MN) are vulnerable to changes in the glial environment is not known, possibly including high excitability with fast, repetitive, and prolonged action potential bursts, high expression of Na^+ channels, high expression of Ca^{2+}-permeable AMPA receptors, high oxidative metabolism, and substantial nitric oxide input (Alvarez and Fyffe, 2007; Carr et al., 2001; Miles, 2000).

The early loss of non-MNs in the present chimeric model suggests the possibility of interneuron-mediated MN degeneration. Spinal cord MNs receive extensive inputs from glycinergic and GABAergic interneurons (Goulding, 2009). Loss of these inhibitory interneurons could potentially lead to hyper-excitability of MNs, which is also observed in cultured MNs that are derived from ALS patient iPSCs (Wainger et al., 2014). Indeed, we observed a significant reduction of GABAergic inputs onto degenerating MNs. Impaired astrocyte function, suggested by low GLT1 expression, and may exacerbate hyper-excitation of MNs, leading to MN degeneration. Our in vivo observation suggests that loss of interneurons play an important role in ALS human astrocyte-induced MN degeneration.

Conclusions

Using the chimera model, we have shown that sALS patient iPSC-astrocytes induce degenerative changes in both MNs and non-MNs of the host, resulting in motor deficits in transplanted mice. Importantly, we have discovered that non-MNs are lost earlier than MNs, with a corresponding reduction of inhibitory nerve terminals in MNs. Thus, the effect of sALS astrocytes on neural degeneration is not specific to MNs, and non-MNs may mediate MN degeneration.

EXPERIMENTAL PROCEDURES

Cell Culture and Differentiation In Vitro

Human spinal neural progenitors were obtained from human ESCs (H9-GFP, passage 21) and iPSCs (the non-ALS IMR-90-4 and sALS iPSC line 1 [JH026] and line 2 [JH028], passages 25–55) after differentiation for 35–45 days in vitro. The transplanted spinal neural progenitors were differentiated according to our previous protocol for spinal MN differentiation (Li et al., 2005). The PSCs were firstly differentiated to neuronepithelia for 8 days, followed by

patterning to ventral spinal progenitors with retinoic acid and purmorphamine from days 10 to 23. The progenitors were then expanded in the presence of basic fibroblast growth factor until days 35 to 45 for transplantation.

Animal Surgery and Cell Transplantation

All procedures were approved by the Animal Care and Use Committee of University of Wisconsin-Madison. The detailed surgical procedure and cell transplantation were described previously (Chen et al., 2015). In brief, adult severe combined immunodeficiency (non-obese diabetic-SCID) mice (aged 8–10 weeks) were obtained from the Jackson Laboratory and anesthetized with 1.5% isoflurane with oxygen and then fixed in a stereotaxic frame. After laminectomy, a total of 5×10^4 neural progenitors of experimental group (in 0.5–1 μL aCSF) or 1 μL aCSF as a sham control were transplanted into the right site of C5-C6 cervical enlargement using a glass micropipette with a tip-diameter of about 50 μm that was attached to the stereotaxic device. The injection site was marked by charcoal before the wound was sutured.

Tissue Preparation

Nine months after cell transplantation, mice were killed with an overdose of pentobarbital (250 mg/kg, intraperitoneally) and perfused with 0.9% sodium chloride followed by 4% paraformaldehyde transcardially. The spinal cord segment was dissected and post-fixed with 4% paraformaldehyde for 4 hr. Following dehydration with 20% and 30% sucrose solution, the specimens were cut into 20-μm-thick consecutive free-floating (cross or sagittal) sections using a microtome.

Immunochemistry and Cell Quantifications

Free-floating sections were rinsed in PBS three times (each time for 10 min) and immersed in blocking buffer (PBS/0.2% Triton X-100/ 10% donkey serum) for 1 hr. The sections were then incubated in primary antibodies (Table S1) overnight at 4°C. After being rinsed in PBS, they were incubated with fluorescein-conjugated secondary antibodies for 1 hr at room temperature. Hoechst 33258 (Sigma) was added for 5 min to label cell nuclei. Images were collected with a Nikon TE600 fluorescence microscope (Nikon Instruments) or a Nikon C1 laser-scanning confocal microscope (Nikon). The populations of human cells (hNu^+), human astrocytes, and GFAP-positive cells were counted in fields randomly selected by an automated stage movement operated by Stereo Investigator software (MicroBrightField) or using z-section images analyzed by ImageJ software (NIH). We selected one section from every 12 sections for each animal, and 6 animals in each group were analyzed for quantification.

ChAT immunoreactivity was used to identify spinal MNs. Only MNs with visible nuclei and nucleolus were selected. MNs with a mean diameter larger than 20 μm (determined by ImageJ) are regarded as putative α-motor neurons (Chang and Martin, 2009). The number of MNs in sections with human astrocytes covering large, medium, and small areas was counted with ImageJ software. The relative intensity of hGFAP and GLT-1 was analyzed using ImageJ software and normalized to the non-ALS group. Punctae of GAD65 and GLYT2 were analyzed using ImageJ software and particles above 0.18 mm^2 were measured according to a previous report (Chang and Martin, 2009). To analyze the NMJs in deltoid muscle, pre-synaptic nerve terminals were detected with a synaptophysin antibody and postsynaptic endplates with BTX. NMJs that had both red (synaptophysin) and green (BTX) staining were considered as innervated, while NMJs stained with only BTX were considered as denervated. Each selected field was imaged at ×20 magnification, at 0.5 μm per step and a z stacked image was generated for each field of view. Ten fields were imaged per deltoid muscle, which corresponds to 100–150 NMJs examined per animal.

Behavioral Tests

In the grip strength test, mice were held to grasp the metered grid with their forepaws, and then gently pulled back horizontally from the base of the tail until their paws withdraw. The peak strength data of forepaws were recorded for five times for each mouse. Gait changes were detected by an unbiased instrument (TreadScan; Columbus Instruments). When the mice walked through the motor-driven treadmill belt at a speed of 11 cm/s for a period of 20 s (Ma et al., 2012), the digital data about footprints and body movement were analyzed by TreadScan software (CleverSys). The data were compared between ipsilateral and contralateral sides and between the transplant and non-transplant groups. The behavioral tests were conducted by a person who was blinded to the experimental groups.

Data Analysis

All the statistical data were presented as mean ± SEM unless otherwise indicated. The differences between groups were compared by Tukey's multiple comparison test in a one-way ANOVA analysis. The significant difference was defined by $p < 0.05$.

SUPPLEMENTAL INFORMATION

Supplemental Information includes three figures and one table and can be found with this article online at http://dx.doi.org/10.1016/j.stemcr.2017.03.003.

AUTHOR CONTRIBUTIONS

K.Q., conception and design, collection and/or assembly of data, data analysis and interpretation, manuscript writing; H.C., conception and design, collection and/or assembly of data, data analysis and interpretation, manuscript writing; A.P., collection of data; H.L.H. and B.Y.H., collection and/or assembly of data, data analysis and interpretation; N.J.M. and G.L.M., collection of sALS patient data and fibroblasts; S.C.Z., conception and design, financial support, collection and/or assembly of data, data analysis and interpretation, manuscript writing, and final approval of manuscript.

ACKNOWLEDGMENTS

This study was supported in part by ALS Association, the Bleser Family Foundation, the Busta Foundation, the NIH (NS076352, NS057778, NS086604, MH099587, and HD076892), the NICHD (P30 HD03352), the National Key Research and Development Project of China (2016YFA0102500), the Innovation Team Cultivation Program of Huazhong University of Science and Technology

(118540263, 118540253, 118540258), and the National Natural Science Foundation of China (81471302). S.C.Z. acknowledges the Steenbock Professorship and is a cofounder of Brainxell, Inc.

Received: October 28, 2016
Revised: March 5, 2017
Accepted: March 6, 2017
Published: March 30, 2017

REFERENCES

Alvarez, F.J., and Fyffe, R.E. (2007). The continuing case for the Renshaw cell. J. Physiol. *584*, 31–45.

Ban, H., Nishishita, N., Fusaki, N., Tabata, T., Saeki, K., Shikamura, M., Takada, N., Inoue, M., Hasegawa, M., Kawamata, S., et al. (2011). Efficient generation of transgene-free human induced pluripotent stem cells (iPSCs) by temperature-sensitive Sendai virus vectors. Proc. Natl. Acad. Sci. USA *108*, 14234–14239.

Bruijn, L.I., Becher, M.W., Lee, M.K., Anderson, K.L., Jenkins, N.A., Copeland, N.G., Sisodia, S.S., Rothstein, J.D., Borchelt, D.R., Price, D.L., et al. (1997). ALS-linked SOD1 mutant G85R mediates damage to astrocytes and promotes rapidly progressive disease with SOD1-containing inclusions. Neuron *18*, 327–338.

Carpenter, S. (1968). Proximal axonal enlargement in motor neuron disease. Neurology *18*, 841–851.

Carr, P.A., Liu, M., and Zaruba, R.A. (2001). Enzyme histochemical profile of immunohistochemically identified Renshaw cells in rat lumbar spinal cord. Brain Res. Bull. *54*, 669–674.

Chang, Q., and Martin, L.J. (2009). Glycinergic innervation of motoneurons is deficient in amyotrophic lateral sclerosis mice: a quantitative confocal analysis. Am. J. Pathol. *174*, 574–585.

Chen, H., Qian, K., Du, Z., Cao, J., Petersen, A., Liu, H., Blackbourn, L.W.T., Huang, C.L., Errigo, A., Yin, Y., et al. (2014). Modeling ALS with iPSCs reveals that mutant SOD1 misregulates neurofilament balance in motor neurons. Cell Stem Cell *14*, 796–809.

Chen, H., Qian, K., Chen, W., Hu, B., Blackbourn, L.W.t., Du, Z., Ma, L., Liu, H., Knobel, K.M., Ayala, M., et al. (2015). Human-derived neural progenitors functionally replace astrocytes in adult mice. J. Clin. Invest. *125*, 1033–1042.

Cirulli, E.T., Lasseigne, B.N., Petrovski, S., Sapp, P.C., Dion, P.A., Leblond, C.S., Couthouis, J., Lu, Y.F., Wang, Q., Krueger, B.J., et al. (2015). Exome sequencing in amyotrophic lateral sclerosis identifies risk genes and pathways. Science *347*, 1436–1441.

Clement, A.M., Nguyen, M.D., Roberts, E.A., Garcia, M.L., Boillee, S., Rule, M., McMahon, A.P., Doucette, W., Siwek, D., Ferrante, R.J., et al. (2003). Wild-type nonneuronal cells extend survival of SOD1 mutant motor neurons in ALS mice. Science *302*, 113–117.

Di Giorgio, F.P., Carrasco, M.A., Siao, M.C., Maniatis, T., and Eggan, K. (2007). Non-cell autonomous effect of glia on motor neurons in an embryonic stem cell-based ALS model. Nat. Neurosci. *10*, 608–614.

Di Giorgio, F.P., Boulting, G.L., Bobrowicz, S., and Eggan, K.C. (2008). Human embryonic stem cell-derived motor neurons are sensitive to the toxic effect of glial cells carrying an ALS-causing mutation. Cell Stem Cell *3*, 637–648.

Goulding, M. (2009). Circuits controlling vertebrate locomotion: moving in a new direction. Nat. Rev. Neurosci. *10*, 507–518.

Gros-Louis, F., Gaspar, C., and Rouleau, G.A. (2006). Genetics of familial and sporadic amyotrophic lateral sclerosis. Biochim. Biophys. Acta *1762*, 956–972.

Haidet-Phillips, A.M., Hester, M.E., Miranda, C.J., Meyer, K., Braun, L., Frakes, A., Song, S., Likhite, S., Murtha, M.J., Foust, K.D., et al. (2011). Astrocytes from familial and sporadic ALS patients are toxic to motor neurons. Nat. Biotechnol. *29*, 824–828.

Hall, E.D., Oostveen, J.A., and Gurney, M.E. (1998). Relationship of microglial and astrocytic activation to disease onset and progression in a transgenic model of familial ALS. Glia *23*, 249–256.

Hayashi, H., Suga, M., Satake, M., and Tsubaki, T. (1981). Reduced glycine receptor in the spinal cord in amyotrophic lateral sclerosis. Ann. Neurol. *9*, 292–294.

Hirano, A., Nakano, I., Kurland, L.T., Mulder, D.W., Holley, P.W., and Saccomanno, G. (1984). Fine structural study of neurofibrillary changes in a family with amyotrophic lateral sclerosis. J. Neuropathol. Exp. Neurol. *43*, 471–480.

Howland, D.S., Liu, J., She, Y., Goad, B., Maragakis, N.J., Kim, B., Erickson, J., Kulik, J., DeVito, L., Psaltis, G., et al. (2002). Focal loss of the glutamate transporter EAAT2 in a transgenic rat model of SOD1 mutant-mediated amyotrophic lateral sclerosis (ALS). Proc. Natl. Acad. Sci. USA *99*, 1604–1609.

Krencik, R., Weick, J.P., Liu, Y., Zhang, Z.J., and Zhang, S.C. (2011). Specification of transplantable astroglial subtypes from human pluripotent stem cells. Nat. Biotechnol. *29*, 528–534.

Li, X.J., Du, Z.W., Zarnowska, E.D., Pankratz, M., Hansen, L.O., Pearce, R.A., and Zhang, S.C. (2005). Specification of motoneurons from human embryonic stem cells. Nat. Biotechnol. *23*, 215–221.

Ma, L., Hu, B., Liu, Y., Vermilyea, S.C., Liu, H., Gao, L., Sun, Y., Zhang, X., and Zhang, S.C. (2012). Human embryonic stem cell-derived GABA neurons correct locomotion deficits in quinolinic acid-lesioned mice. Cell Stem Cell *10*, 455–464.

Marchetto, M.C., Muotri, A.R., Mu, Y., Smith, A.M., Cezar, G.G., and Gage, F.H. (2008). Non-cell-autonomous effect of human SOD1 G37R astrocytes on motor neurons derived from human embryonic stem cells. Cell Stem Cell *3*, 649–657.

Martin, L.J., and Chang, Q. (2012). Inhibitory synaptic regulation of motoneurons: a new target of disease mechanisms in amyotrophic lateral sclerosis. Mol. Neurobiol. *45*, 30–42.

Martin, L.J., Liu, Z., Chen, K., Price, A.C., Pan, Y., Swaby, J.A., and Golden, W.C. (2007). Motor neuron degeneration in amyotrophic lateral sclerosis mutant superoxide dismutase-1 transgenic mice: mechanisms of mitochondriopathy and cell death. J. Comp. Neurol. *500*, 20–46.

Maruyama, H., Morino, H., Ito, H., Izumi, Y., Kato, H., Watanabe, Y., Kinoshita, Y., Kamada, M., Nodera, H., Suzuki, H., et al. (2010). Mutations of optineurin in amyotrophic lateral sclerosis. Nature *465*, 223–226.

Meyer, K., Ferraiuolo, L., Miranda, C.J., Likhite, S., McElroy, S., Renusch, S., Ditsworth, D., Lagier-Tourenne, C., Smith, R.A., Ravits, J., et al. (2014). Direct conversion of patient fibroblasts demonstrates non-cell autonomous toxicity of astrocytes to motor

neurons in familial and sporadic ALS. Proc. Natl. Acad. Sci. USA *111*, 829–832.

Miles, R. (2000). Perspectives: neurobiology. Diversity in inhibition. Science *287*, 244–246.

Nagai, M., Re, D.B., Nagata, T., Chalazonitis, A., Jessell, T.M., Wichterle, H., and Przedborski, S. (2007). Astrocytes expressing ALS-linked mutated SOD1 release factors selectively toxic to motor neurons. Nat. Neurosci. *10*, 615–622.

Petri, S., Krampfl, K., Hashemi, F., Grothe, C., Hori, A., Dengler, R., and Bufler, J. (2003). Distribution of GABAA receptor mRNA in the motor cortex of ALS patients. J. Neuropathol. Exp. Neurol. *62*, 1041–1051.

Re, D.B., Le Verche, V., Yu, C., Amoroso, M.W., Politi, K.A., Phani, S., Ikiz, B., Hoffmann, L., Koolen, M., Nagata, T., et al. (2014). Necroptosis drives motor neuron death in models of both sporadic and familial ALS. Neuron *81*, 1001–1008.

Schiffer, D., Cordera, S., Cavalla, P., and Migheli, A. (1996). Reactive astrogliosis of the spinal cord in amyotrophic lateral sclerosis. J. Neurol. Sci. *139*, 27–33.

Serio, A., Bilican, B., Barmada, S.J., Ando, D.M., Zhao, C., Siller, R., Burr, K., Haghi, G., Story, D., Nishimura, A.L., et al. (2013). Astrocyte pathology and the absence of non-cell autonomy in an induced pluripotent stem cell model of TDP-43 proteinopathy. Proc. Natl. Acad. Sci. USA *110*, 4697–4702.

Wainger, B.J., Kiskinis, E., Mellin, C., Wiskow, O., Han, S.S., Sandoe, J., Perez, N.P., Williams, L.A., Lee, S., Boulting, G., et al. (2014). Intrinsic membrane hyperexcitability of amyotrophic lateral sclerosis patient-derived motor neurons. Cell Rep. *7*, 1–11.

Wang, L., Gutmann, D.H., and Roos, R.P. (2011). Astrocyte loss of mutant SOD1 delays ALS disease onset and progression in G85R transgenic mice. Hum. Mol. Genet. *20*, 286–293.

Whitehouse, P.J., Wamsley, J.K., Zarbin, M.A., Price, D.L., Tourtellotte, W.W., and Kuhar, M.J. (1983). Amyotrophic lateral sclerosis: alterations in neurotransmitter receptors. Ann. Neurol. *14*, 8–16.

Windrem, M.S., Schanz, S.J., Guo, M., Tian, G.F., Washco, V., Stanwood, N., Rasband, M., Roy, N.S., Nedergaard, M., Havton, L.A., et al. (2008). Neonatal chimerization with human glial progenitor cells can both remyelinate and rescue the otherwise lethally hypomyelinated shiverer mouse. Cell Stem Cell *2*, 553–565.

Wong, P.C., Pardo, C.A., Borchelt, D.R., Lee, M.K., Copeland, N.G., Jenkins, N.A., Sisodia, S.S., Cleveland, D.W., and Price, D.L. (1995). An adverse property of a familial ALS-linked SOD1 mutation causes motor neuron disease characterized by vacuolar degeneration of mitochondria. Neuron *14*, 1105–1116.

Yamanaka, K., Chun, S.J., Boillee, S., Fujimori-Tonou, N., Yamashita, H., Gutmann, D.H., Takahashi, R., Misawa, H., and Cleveland, D.W. (2008). Astrocytes as determinants of disease progression in inherited amyotrophic lateral sclerosis. Nat. Neurosci. *11*, 251–253.

Yu, P., Matloub, H.S., Sanger, J.R., and Narini, P. (2001). Gait analysis in rats with peripheral nerve injury. Muscle Nerve *24*, 231–239.

Stem Cell Reports, Volume *8*

Supplemental Information

Sporadic ALS Astrocytes Induce Neuronal Degeneration In Vivo

Kun Qian, Hailong Huang, Andrew Peterson, Baoyang Hu, Nicholas J. Maragakis, Guo-li Ming, Hong Chen, and Su-Chun Zhang

Supplemental Figures and legends

Figure S1. Characteristics of pluripotent stem cells, related to Fig1. (A) A representative phase contrast image of an iPSC colony. Scale bar=50μm. (B-F) The iPSCs were stained for alkaline AKP (C), SOX2 (D), NANOG (E), OCT4 (F) and SSEA4 (G). Scale bar=50μm. (G-I) Representative sections of teratomas formed by the iPSCs showing ectoderm (H), mesoderm (I), and endoderm (J) tissues. Scale bar=50μm. (J) Karyotypes of sALS-1 and sALS-2 iPSCs at passage 40.

Figure S2. Integration of non-ALS and sALS human astrocytes into the adult spinal cord, related the result part '*Neural cells from sALS iPSCs integrate into the adult mouse spinal cord*'. (A) Adjacent sections of the transplanted spinal cord showing distribution and overlap (arrows) of $hGFAP^+$ cells (left panel) and hNu^+ cells (right panel). The boxed area is magnified on the left corner. (B, C) Cross sections showing relationship between $hGFAP^+$ human astrocytes and $ChAT^+$ MNs or $Map2^+$ neurons for non-ALS (B) and ALS (C) groups in the gray matter. (D, E) Immunostaining shows relationship between $hGFAP^+$ human astrocyte processes and $laminin^+$ blood vessels in non-ALS (D) and sALS (E) groups. Scale bar=50μm.

Figure S3. Ubiquitin inclusions in $ChAT^+$ MNs in different segments of the transplanted spinal cord, related to Fig 4B. (A, B) Staining for ubiquitin showing ubiquitin inclusions (arrows) in $ChAT^+$ MNs in the spinal cord transplanted with sALS cells (B) and non-ALS cells (A). The transplanted cells were marked by hGFAP. The arrows indicate ubiquitin inclusions on the $ChAT^+$ cell body. Scale bar=50μm.

Fig S1

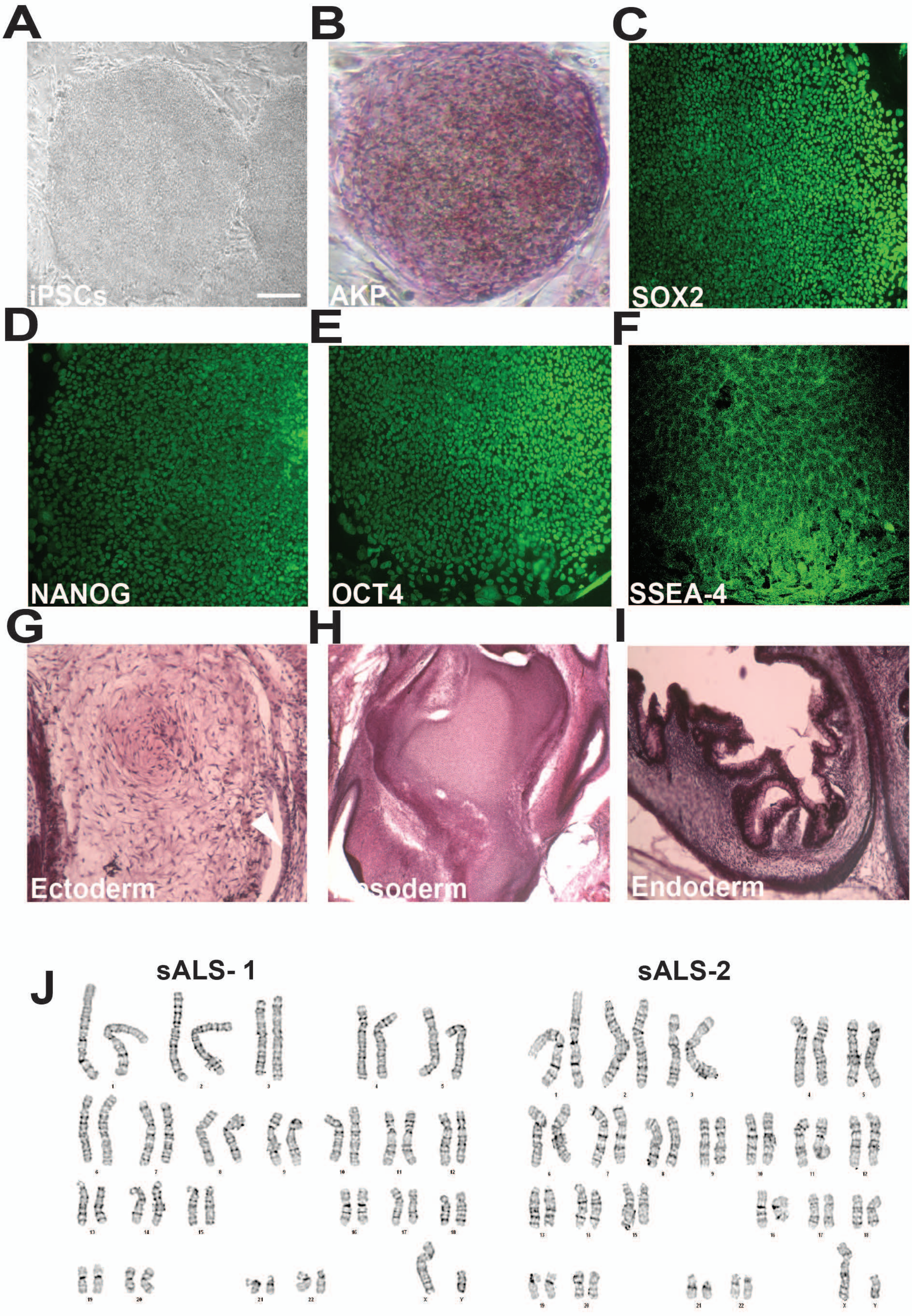
A
B
C
iPSCs
AKP
SOX2
D
E
F
NANOG
OCT4
SSEA-4
G
H
I
Ectoderm
soderm
Endoderm
J
sALS- 1
sALS-2

Fig S2

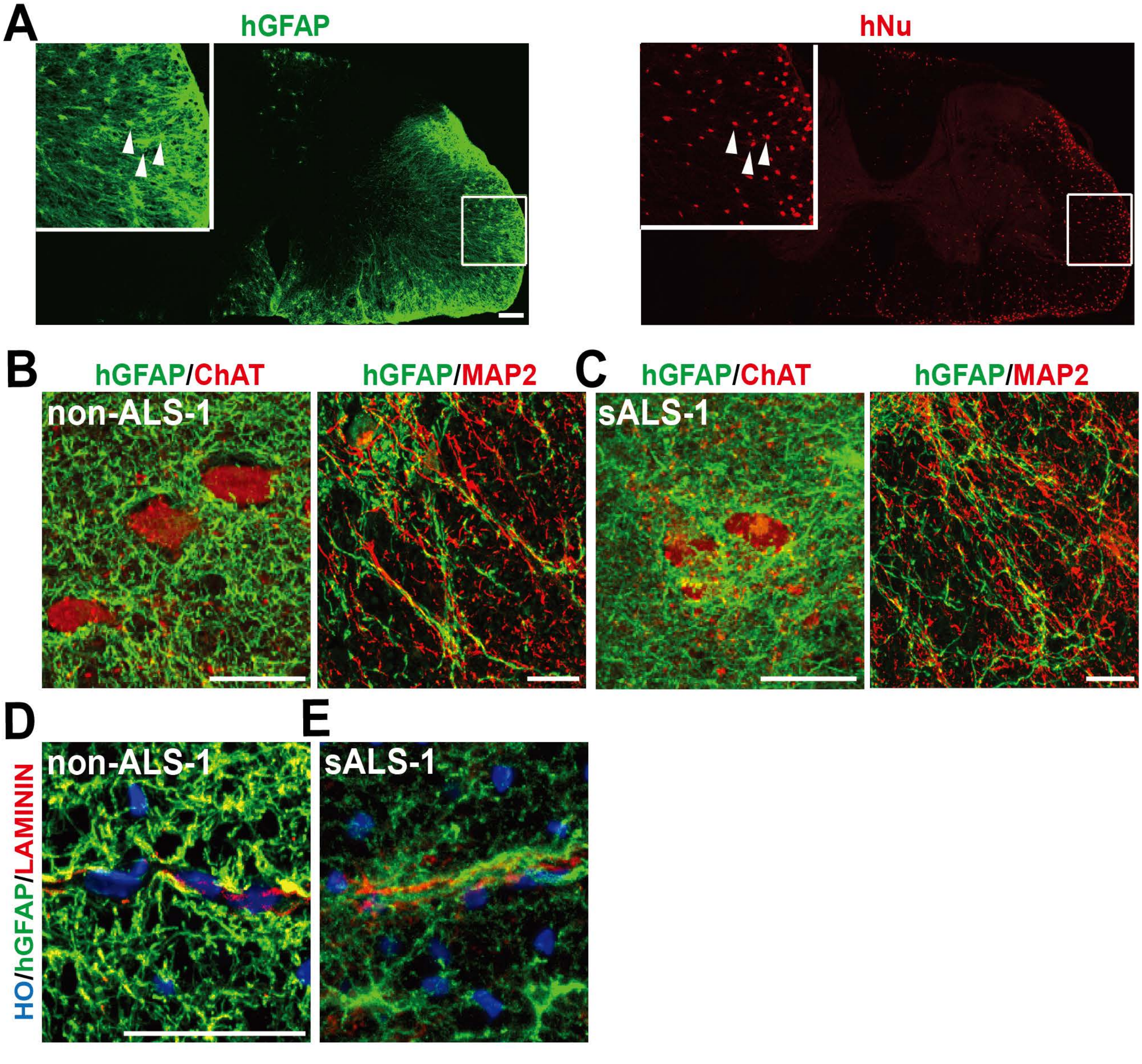

A
hGFAP
hNu
B
hGFAP/ChAT
non-ALS-1
hGFAP/MAP2
C
hGFAP/ChAT
sALS-1
hGFAP/MAP2
D
non-ALS-1
HO/hGFAP/LAMININ
E
sALS-1

Fig S3

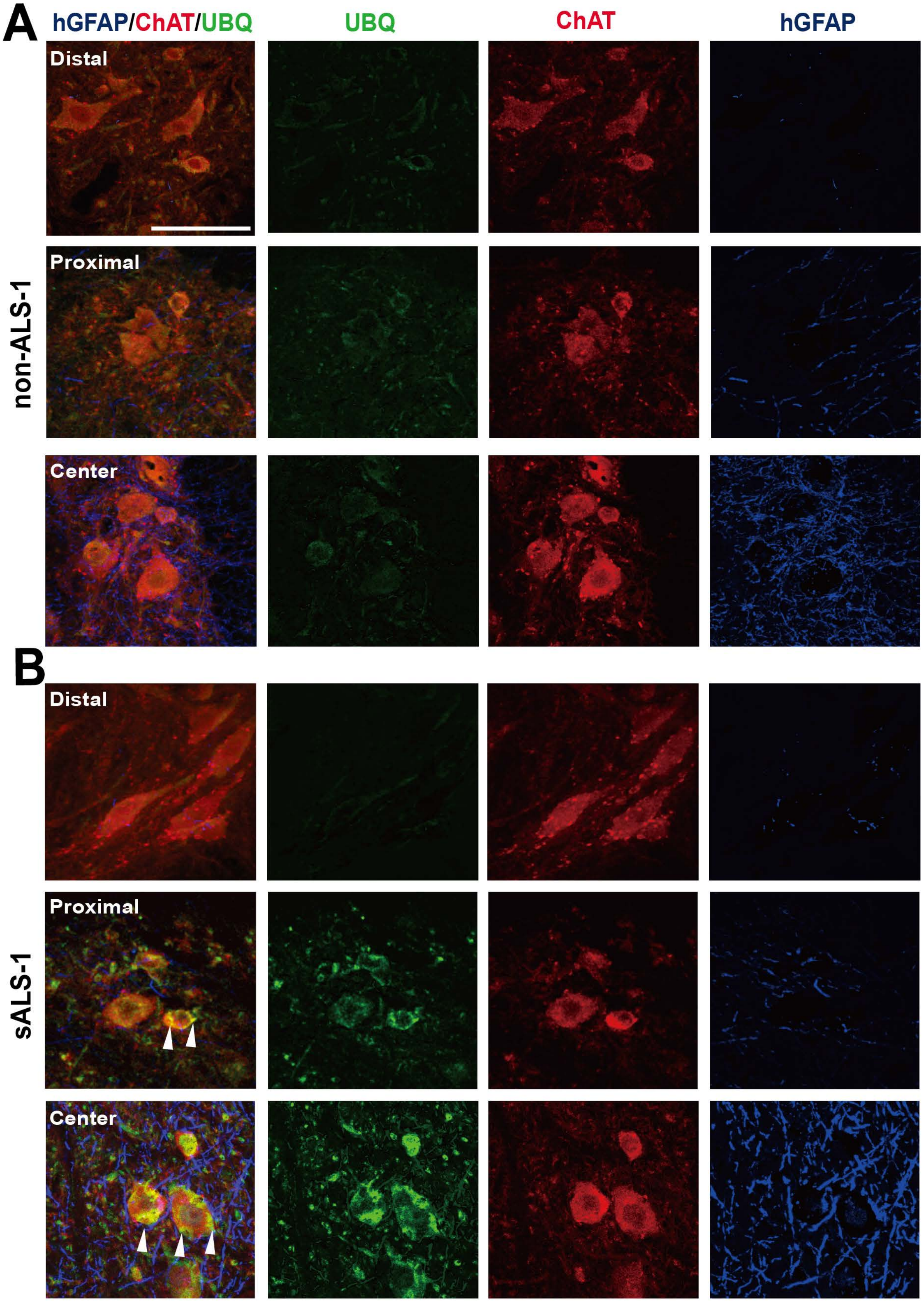
A
hGFAP/ChAT/UBQ
UBQ
ChAT
hGFAP
Distal
Proximal
Center
non-ALS-1
B
Distal
Proximal
Center
sALS-1

Table S1 the list of antibodies used in the study, related to all figures.

Antibody	Isotype	Dilution	Source (Catalog no.)
BTX-488		1:500	Life technologies (B13422)
ChAT	Goat IgG	1:300	Chemicon & Millipore (AB144P)
GFAP	Rabbit IgG	1:5000	DAKO (Z0334)
GAD65	Rabbit IgG	1:2000	Chemicon & Millipore (AB1511)
Human Nuclei	Mouse IgG	1:200	Chemicon & Millipore (MAB1281)
hGFAP	Mouse IgG	1:500	Stem Cells, Inc (AB-123-U-050)
Neurofilament 200	Rabbit IgG	1:1000	Sigma (N4142)
TUBULIN	Rabbit IgG	1:4000	Chemicon & Millipore (AB9354)
MAP2	Rabbit IgG	1:5000	Chemicon & Millipore (AB5622)
NeuN	Rabbit IgG	1:500	Chemicon & Millipore (ABN78)
OLIG2	Rabbit IgG	1:500	Chemicon & Millipore (AB9610)
OTX2	Goat IgG	1:2000	R&D (AF1979)
HOXB4	Rat IgG	1:50	DSHB (I12 anti-Hoxb4)
SYNAPTOPHYSIN	Mouse IgG	1:1000	Chemicon & Millipore (MAB5258)
GLT-1	Guinean pig IgG	1:20000	Chemicon & Millipore (AB1783)
GLYT2	Guinean pig IgG	1:1000	Chemicon & Millipore (AB1773)
UBIQUITIN	Rabbit IgG	1:1000	DakoCytomation (Z0458)
